# Deaths of patients diagnosed with psychotic disorder due to SARS Cov-2 in Avilés, Spain

**DOI:** 10.1192/j.eurpsy.2024.1048

**Published:** 2024-08-27

**Authors:** M. A. Reyes Cortina, L. Pérez Gómez, L. Iglesias Fernández, R. Fernandez García, J. J. Martínez Jambrina

**Affiliations:** ^1^Mental Health, Hospital Universitario San Agustin, Aviles; ^2^Mental Health, Hospital Universitario San Agustín, Avilés, Spain

## Abstract

**Introduction:**

Psychotic patients are a vulnerable population from a social and health point of view. The SARS Cov-2 pandemic affected millions of people around the world, however, its effects on psychotic patients in Avilés Spain, have not been analized.

**Objectives:**

The objective of this study was to determine and compare the mortality of patients with psychosis due to SARS Cov-2 in Avilés, Spain with others regions and countries in the European Union. Determine the influence of social condition and antipsychotic treatment on the condition of these patients.

**Methods:**

This is a descriptive, observational study, in which patients diagnosed with psychosis in the period 2020-2021 who contracted SARS Cov-2 infection in Avilés, Spain, were studied to determine those who died from this cause. The influence of social status and antipsychotic medication, as well as sociodemographic factors (age, sex, marital status) were analyzed and compared with other regions and countries of the European Union.

**Results:**

Despite the high mortality rate in patients with psychosis, during the years of the pandemic SARS Cov-2 played an important role given the vulnerability of these patients.

**Conclusions:**

The negative effects and deaths during the COVID-19 pandemic were at the time a major problem for public health worldwide. This study concluded that the morbidity and mortality of psychotic patients who contracted COVID-19 was lower than the rest of the population.

**Disclosure of Interest:**

None Declared

